# Conceptualization of Cloud-Based Motion Analysis and Navigation for Wearable Robotic Applications

**DOI:** 10.3390/s24154997

**Published:** 2024-08-02

**Authors:** David Schick, Johannes Schick, Jonas Paul David, Robin Neubauer, Markus Glaser

**Affiliations:** Institute for High Integrity Mechatronics Systems, Aalen University, 73430 Aalen, Germany; david.schick@hs-aalen.de (D.S.);

**Keywords:** human activity recognition, navigation, map construction, active orthosis, exoskeleton

## Abstract

The behavior of pedestrians in a non-constrained environment is difficult to predict. In wearable robotics, this poses a challenge, since devices like lower-limb exoskeletons and active orthoses need to support different walking activities, including level walking and climbing stairs. While a fixed movement trajectory can be easily supported, switches between these activities are difficult to predict. Moreover, the demand for these devices is expected to rise in the years ahead. In this work, we propose a cloud software system for use in wearable robotics, based on geographical mapping techniques and Human Activity Recognition (HAR). The system aims to give context to the surrounding pedestrians by providing hindsight information. The system was partially implemented and tested. The results indicate a viable concept with great extensibility prospects.

## 1. Introduction

### 1.1. Motivation

The demographic change in Germany poses a challenge to society. Predictions show that in 2060, up to 47% of the population will be aged 65 or higher [1,2] indicating a growing demand on elder care. This situation is similar for many industrial nations [3], and in the case of Japan, it is even worse [4]. Lower-limb exoskeletons and active orthoses have been thoroughly discussed in the literature as assistive devices [5,6,7]. As practical examples, they are used in the field of rehabilitation in stroke recovery and assisting patients with partial gait disabilities or age-dependent frailty [6,7]. However, for the devices to find widespread use, patients need to be able to use these devices independently outside of a clinical environment. Usability and safety pose a challenge which is yet to be solved.

An active exoskeleton needs to support activities of daily living. Arguably, among the most important activities are level walking and climbing stairs. The exoskeleton must be aware of the executed movement task to correctly assist the user. For example, handheld switches have been used to select different movement tasks manually [8,9]. This solution constrains the user (who must be able to reach the switches) and is prone to user errors. Efforts have been made to solve this issue by enabling exoskeletons to autonomously discern different movement tasks. Attempts include the use of cameras for image recognition [10,11] and machine learning algorithms [12,13]. Still, safely identifying or even predicting the user intention remains a challenge, especially in the context of medical devices and the resulting regulatory requirements [14].

In this work, an approach to support the safe identification of movement tasks is presented. The approach includes a navigable map which contains information on previously encountered movement tasks. This map is intended to be generated automatically using Human Activity Recognition (HAR) in combination with an iterative map construction algorithm. A user intent recognition algorithm can then use the information on this map, for example, to constrain predictions based on previously encountered activities.

### 1.2. Related Work

In our previous publication SensAA, a mobile, cloud-based biomechanical data acquisition system was introduced [15]. The SensAA system is used in this work to generate datasets and verify the solution. Furthermore, we will extend the system’s cloud server capabilities to support the storage and analysis of geospatial information.

#### 1.2.1. Human Activity Recognition

Human Activity Recognition (HAR) aims at recognizing different activities conducted by humans based on measurements taken from the humans’ motion. HAR is a multiclass classification problem, typically implemented using various machine learning algorithms. Lara et al. [16] published a survey paper giving a comprehensive overview of research on HAR systems using wearable sensors. They identified a common architecture in data acquisition systems, consisting of wearable sensors, an interface device, and optionally a server environment for storage and analysis. They also evaluated different models, including Support Vector Machines (SVMs), Decision Trees, and Deep Learning Models. Good accuracies were found with multiple models, including SVMs (~90%) and Decision Trees (~85%). Note that they also considered non-walking activities, like cooking and bike-riding. When only walking activities were considered, the accuracy was even higher. Jordao et al. validated different models for HAR problems on publicly available datasets [17]. They found two families of models commonly used: some models use hand-crafted features, while other models, i.e., Convolutional Neural Networks, perform the feature extraction automatically by fitting on the training set. The most popular method for automatic feature extraction in recent publications is Convolutional Neural Networks (CNN) [18,19,20,21]. In Jordao’s comparison, a CNN-based model achieved the highest overall accuracy, but without significantly better results compared to well-selected handcrafted features [17]. Their benchmark results also indicate that CNN approaches are more prone to overfitting, since the results of only one of the four reviewed CNN models could be reproduced when verified. Nevertheless, with recent advances, CNN-based approaches are gaining popularity in recent years. Self-attention-based methods take advantage of the sequential nature of gait information to achieve more robust classifications [22,23,24]. Methods used for self-attention include LSTM (Long Short-Term Memory) [23] and GRU (Gated recurrent units) [24]. Mutegeki et al. proposed a CNN-LSTM model for human activity recognition. Their classifier scored a 92% accuracy on the UCI public HAR dataset [25] containing accelerometry data acquired from smartphones. They state that their model, while increasing the accuracy, is computationally less complex when compared to a conventional CNN model [23].

#### 1.2.2. Map Construction

Two common approaches in map construction are the “raster map” and the “vector map”. Raster maps are stored as a grid of pixels, while a vector map stores the geometries as vector information [26]. Raster maps allow for faster computations of many spatial analysis methods, but only vector maps can store geometries, like a roadway, accurately [26,27]. A vector map can be converted to a raster map by rendering it into an image. In contrast, deriving vector data from raster data requires complex calculations [28]. Constructing maps from GPS traces has already been thoroughly discussed. However, most algorithms are designed to recreate roadways from GPS traces recorded by cars. Bruntrup et al. proposed such an algorithm [29]. They subdivided a map into tiles for faster access and used machine learning methods to infer the road geometry. The road was stored as vector information [29]. Zhang et al. proposed a map construction algorithm for creating road maps from GPS traces [30]. In contrast to Bruntrup’s algorithm, their map construction algorithm creates a raster map. The key feature of their method is that the raster map can be compressed to lower the resolution while still maintaining topological correctness [30]. Kasemsuppakorn et al. [31] developed an algorithm for automatically generating a network from GPS traces for paths used by pedestrians. In their work, they mainly focused on sidewalks. The network was generated using self-collected GPS traces and publicly available traces from the Open Street Maps (OSM) project. The algorithm was evaluated using the metrics of geometrical correctness and topological correctness as proposed by Wiedemann et al. [32]. The evaluation resulted in an average geometrical correctness of 57.55% and an average topological correctness of 76.9%.

## 2. Materials and Methods

### 2.1. System Architecture

**Figure 1:**
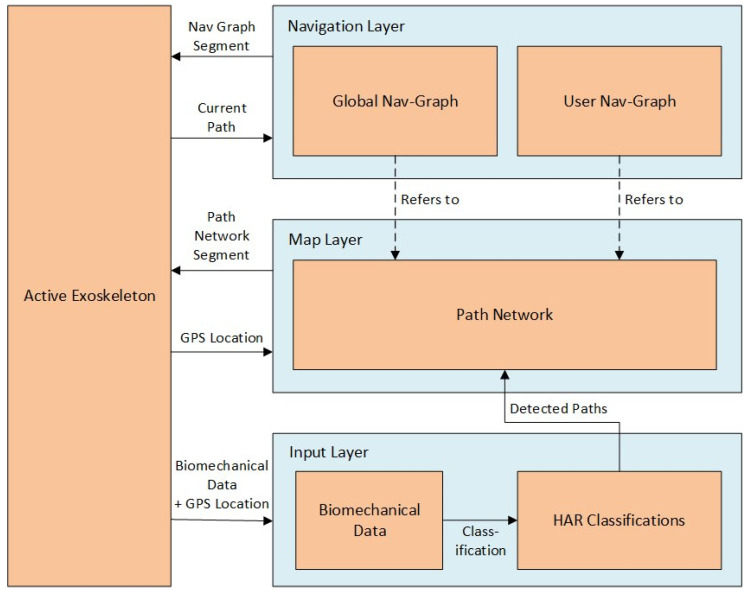
System architecture.

The architecture of the proposed system includes three layers of data, with an increased abstraction from bottom to top. A block diagram of the architecture is shown in Figure 1.

From bottom to top, the first layer is the input layer. It contains raw sensor information as well as classification results of individual steps. The data of the input layer is supplied by the sensors located on the exoskeleton. The data is then split into steps and classified by an HAR algorithm to determine the type of activity. We keep the data in the input layer to make the resulting map reproducible.

The second layer is the map layer. The map layer contains the path network, which is continuously updated from sequences of steps from the input data layer. Paths have different types assigned to them, based on the determined movement activity of the step. These paths are connected by crossings. Crossings and paths can be interpreted as nodes and edges of a bidirectional graph.

The navigation layer forms the top layer of the system architecture and memorizes which paths users move on through the path network. This information is split into a global navigation graph and a user-local graph. The local navigation graphs are iteratively merged into the global navigation graph. The global navigation graph can be used by the user to gain insight into areas which were not frequented by the user themself. The local navigation graph, on the other hand, holds personalized navigation information for the user.

### 2.2. Human Activity Recognition

Path types in the map are identified based on Human Activity Recognition (HAR) from biomechanical data, which can be described as a multivariant classification problem of a time series signal. The activities to be classified define the classification targets. For our problem, we need to distinguish the following gait activities:

- Level walking;
- Stairs ascending;
- Stairs descending.

A classification model consists of two major parts. First, the raw input data must be pre-processed to generate samples from the time series. Then, a classifier assigns one of the trained targets to the sample. The raw input data consists of multiple signals, recorded with two IMU (Inertial Measurement Unit) sensors mounted at the thigh and shank of the subject as follows:

- Inclination of the shank and thigh in the sagittal plane;
- Angular velocities (X, Y, Z) of the shank and thigh;
- Acceleration (X, Y, Z) of the shank and thigh.

The pre-processing pipeline generates samples from the input data in three steps. In the first step, the time series signals must be divided into time windows. These windows represent the discrete time frames the classifier is using at a given time. From the data in these windows, the features are extracted. The last step of preprocessing is scaling. The scaler scales the feature values onto values between zero and one. This step should avoid unwanted differences impacting individual features.

Since the classifications are all human gait activities, the data to classify is a periodic signal. Many statistical features like minimum and maximum values, can have a higher impact when classifying the signal per period. One period of the signal corresponds to one gait cycle. A step detection algorithm, observing only one leg, is used to find the beginning of each cycle. To detect the steps, an algorithm similar to the one proposed by Diaz et al. is used. The method uses the pitch angle of the thigh as its only input signal [33]. When the angle is at its maximum, the subject’s heel is close to striking the ground, which is the beginning of a step. Since only a separation of the signal into periodic windows is needed, this inaccuracy can be accepted, which also allows for a faster implementation. To avoid detecting a long standing period between two steps as a gait cycle, we assume a gait cycle to be no longer than two seconds. Thus, every period between two detected steps with a length of less than two seconds is assumed to be a step and forms a time window. In this work, we only observe the movement of one leg. This implies that one step in our classification means a full stride of this leg. The step-detector was implemented using the peak detection algorithm contained in the ‘signal’ package of the ‘scipy’ python library. The peak detection works by first finding all local maximums and then filtering them by different properties. To detect the maximum inclination as a peak, the following parameters were used:

- Prominence: 0.2;
- Height: 1.6 radians;
- Distance: 16 samples.

Features are extracted from time windows to reduce the dimensionality of the samples. This process was developed in two steps. First, a literature review on potentially useful candidate features was conducted. Then, the features were tested for correlations with the target classes. The features that correlated the most with the target were selected for the feature vector. The features finally selected are:

- Minimum values of shank and thigh inclination;
- Maximum values of shank and thigh inclination;
- Mean values of shank and thigh inclination;
- Standard deviation of shank and thigh inclination;
- Range between minimum and maximum values of shank and thigh inclination.

Different classifiers were evaluated. After comparing the performance of a simple decision tree with a Support Vector Machine (SVM) classifier, an SVM classifier in a one-vs-rest configuration was chosen. The full model evaluation is depicted in Section 3.1.

### 2.3. Map Construction

When generating a geographically unrestricted map, large amounts of data accumulate over time. At this point, resampling the entire map every time new parts are inserted quickly becomes unfeasible. An iterative map construction strategy, on the other hand, can be applied to just a part of the map, which is why such an algorithm was used in this project. Our map contains the paths the subject walks during the day. These paths are interconnected at crossings. Each path can have additional properties, since different facilities, like stairs, will be used by the subject. The following sections describe the map construction system. First, the data model representing the map is described. Then, the operations which can be applied to the path model are outlined. These operations form the building blocks comprising the algorithm. Finally, the algorithm realizing the insertion-based map construction is described.

#### 2.3.1. Data Model

**Figure 2:**
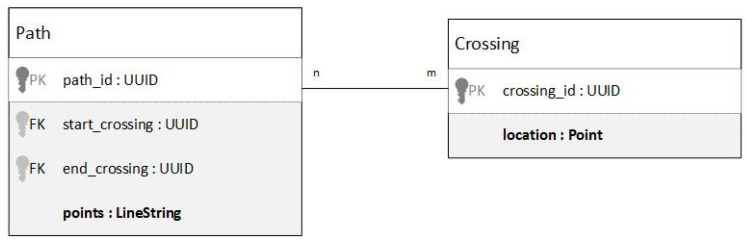
ER model of the map.

The data model must be capable of holding the entire map while being able to quickly retrieve parts of it. To persistently save the data model, a Relational Database optimized for geospatial operations is used. There are several options available; for this project, the PostGIS extension version 3.4 for the PostgreSQL Database Management System (DBMS) version 15 was used. To store the map, a database schema must be defined. To define the schema, entities and their relationships are modeled using a UML (Unified Modeling Language) Entity Relationship (ER) diagram. Figure 2 shows the ER diagram for the map.

The ‘path’ entity holds a single path. We use special spatial datatypes provided by the database to define the actual path, which is a sequence of geographical points. A path can, both at the start and the end, be connected to many other paths via a crossing. The connection is determined by the ID of the crossing. In this way, two n-to-m relationships between paths and crossings are implemented: one for connections to the start point of a path, and one for connections to the end point. The location of a crossing can be implicitly determined by looking at the location of the start point and the respective end point of the connected paths. However, by storing the location of a crossing in the crossing entity itself, crossings can be looked up via their location.

#### 2.3.2. Path Operations

To enable the creation of an iterative map construction algorithm, a few operations for manipulating paths must be implemented. To gain a better understanding of which operations are necessary, we first defined how we want the map to change when new paths are inserted, and how this affects the existing paths in the map. When inserting a path, any part of the new paths matching an existing path is used to update the existing path. Any non-matching part is added to the map, with a crossing at the spot where the matching and non-matching part meet. This involves multiple operations, many of which manipulate the path’s geometry and properties:

- Paths must be matched with other paths, to find points in which they start matching and stop matching. This operation is called ‘Path matching’.
- Paths must be split into multiple segments at arbitrary points, and between vertices. This operation is called ‘Path splitting’. When splitting paths, some of the properties must be recalculated. For example, if a path of type stair is split at its center, the ‘number of stairs’ property is divided by two.
- Paths can be simplified. This involves splitting the paths to remove loops and down sampling paths with an excessive density of vertices.
- Paths must be merged with matching paths to form an updated and potentially more accurate path. This operation is called ‘Path merging’. When merging two paths, a weighted average is applied to the geometry and the path properties. The weight of the average is proportional to the number of merges a path has made.

Additionally, paths support general geometrical operations, like calculating their length, finding their centroid, or calculating the bounding box. Since these calculations are trivial, they are not described in detail in this article.

#### 2.3.3. Map Construction Algorithm

**Figure 3:**
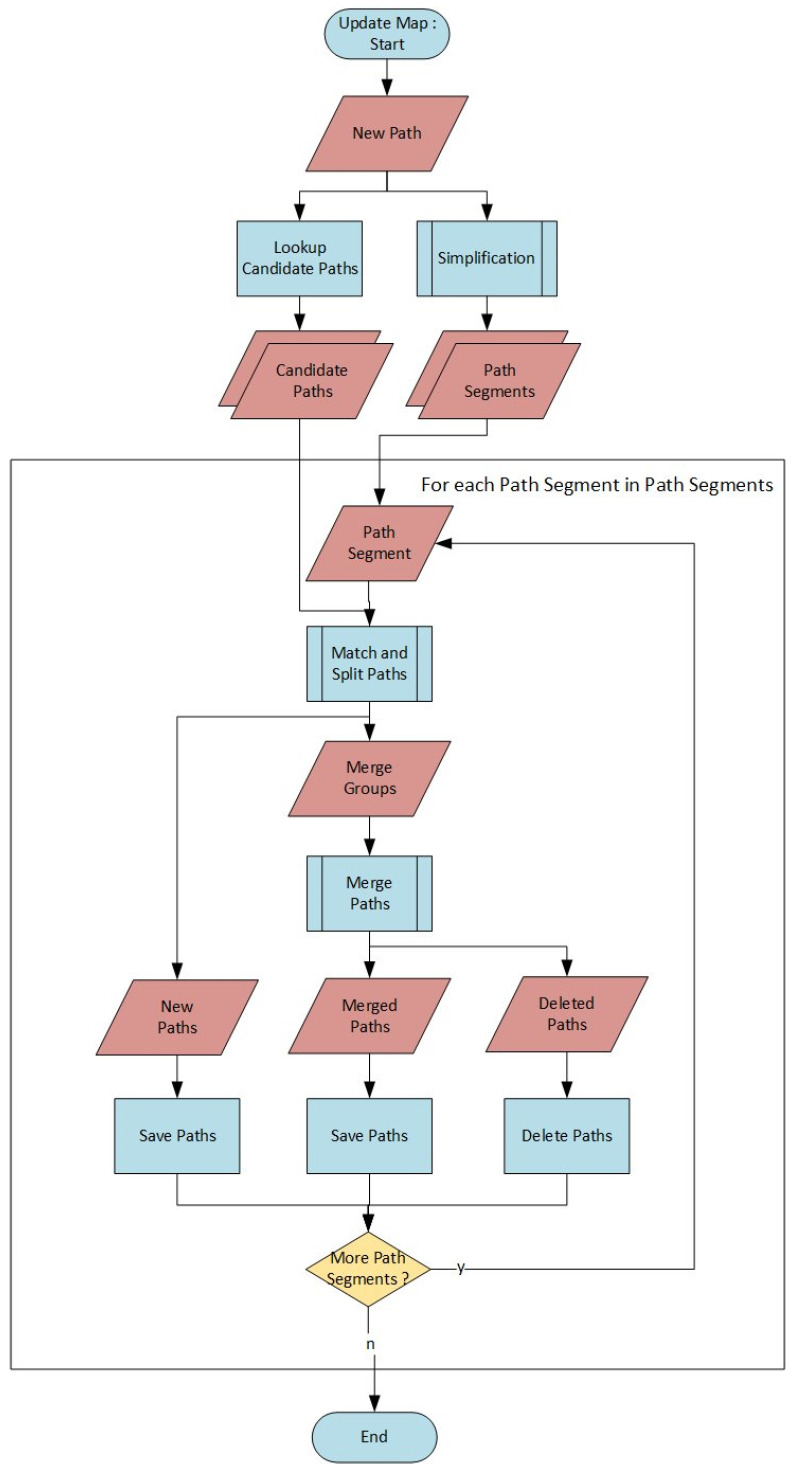
Map construction algorithm.

The map construction algorithm is iteratively applied to each path added to the map. The flowchart in Figure 3 shows the algorithm. Since the algorithm cannot insert paths that contain loops, the path is first simplified. Simplification results in one or more non-self-intersecting path segments. Then, all paths which might be matching the new paths (candidate paths) are looked up. The lookup is performed by filtering all paths that intersect with the new path’s bounding box. Now, the map insertion algorithm is applied to all simplified path segments. Each path segment is matched with the candidate paths. The matching segments are split into groups of matching paths (merge groups) and non-matching paths. Non-matching segments of the new path have not yet been made part of the map. They are inserted into the map as new paths. The merge groups are combined (merged) using a weighted average of the number of times the paths were previously merged. After merging, a new path results from the combination of the two matching segments. The two input paths are no longer needed and are deleted.

Whenever paths are matched and merged, a gap between non-matching and matching parts appears. This gap is closed by inserting a connector, outlining a connection between the matching and non-matching part. If a path is matched from its beginning or end, the connector at this end is kept, resulting in a crossing of four or more paths.

## 3. Results

The system has been partly implemented and verified. First, the HAR classifier, determining the activity state of the user, has been verified. Then, the map construction algorithm was tested with GPS traces recorded by a cellphone. The HAR classifier and the map construction algorithm will be integrated in a later stage of the project. Therefore, we consider all paths as simple trails in this verification.

### 3.1. Human Activity Recognition

**Figure 4:**
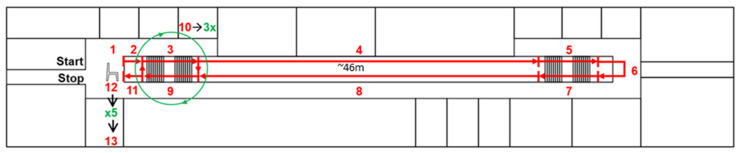
Schematic of the track used for acquisition of datasets. The numbers indicate the succession of the track.

The performance of the classifier was verified using a dataset recorded by SensAA, a wearable, IMU-based biomechanical data acquisition system introduced in our previous publication [15] The system provides three-axis acceleration and angular velocity measurements. In addition, the inclination of the sensor in the sagittal plane is recorded. The average sampling rate is ~30 Hz. The dataset was recorded indoors at Aalen University, at a long doorway that included two staircases. Figure 4 shows the track used for data acquisition. The track consists of two staircases and a 46 m long hallway. Both staircases consist of 21 steps. Each staircase is separated into two stairs. The upper staircase has 11 steps, and the lower staircase has 10 steps. The steps are divided by a 1.6 m wide plateau. 

Each subject started at the top of the staircase (2), shown on the left in the schematic. Then, the subject walked down the staircase (3), along the hallway (4), and up the staircase at the end of the hallway (5). From the top of the staircase (6), the subject walked the same way back to the starting point (7–11). Then, the subject walked the staircase from the starting position three times up and down (10) to obtain sufficient samples of the stairs-ascending and stairs-descending classes. At the end of the track, the subject was standing up and then sitting down on a chair five times to obtain samples of standing-up and sitting-down activities (12). However, these classes were not used in this work. After the recording, the datasets were labeled manually by marking the timeframes of the activities. To find the start and end of the activities, the signal was manually viewed and compared to literature data. Over these timeframes, the preprocessing pipeline described in Section 2.2 was applied to generate labeled samples.

The dataset contains 46 recorded trials of 22 different subjects. The group of subjects consists of 15 males and 7 females. The subjects can be divided into two age groups, with 7 subjects aged between 20 and 30 years, and 15 subjects in the group of 50 to 70 years. All subjects gave their verbal informed consent before the trials. Despite our best effort, neither the age, nor the physical properties of our subjects were equally distributed throughout the dataset. To account for the potential bias introduced by the set of subjects, two strategies for selecting training and test datasets were applied. The leave-one-subject-out verification uses the trials recorded by one subject as the test dataset, while using the trials of all other subjects as the training dataset. The leave-one-trial-out verification forms the test dataset from one trial of one subject, while using all trials of all other subjects as the training dataset. Both verifications were repeated for all subjects in the respective trials. For each run, the average precision, accuracy, and F1 scores were calculated over all targets. The results include the minimum, maximum, and average values of these metrics over all runs. Since the verification was also conducted for model selection, a Decision Tree with a maximum branch depth of three was implemented. The performance between the Decision Tree model and the Support Vector Machine (SVM) model was compared. Table 1 shows the results of the verification. The results indicate a high overall accuracy, precision, and F1 score of more than 96% for both the SVM and the Decision Tree classifier. This indicated that both models were suitable for the use case. While the mean accuracy, precision, and F1 score were practically equal between both models (±0.1%), the SVM model showed a slightly smaller standard deviation of the results (0.05–0.09%) compared to the Decision Tree Model (0.03–0.11%) in the leave-one-subject out verification. In the leave-one-trial out verification, the results were almost identical. The decision tree model with a depth of only three branches evaluated than the SVM model. Since the system is deployed on a server with practically no energy efficiency requirements, and the SVM outperformed the Decision Tree ever so slightly, the SVM model was adopted in this project.

**Figure 5:**
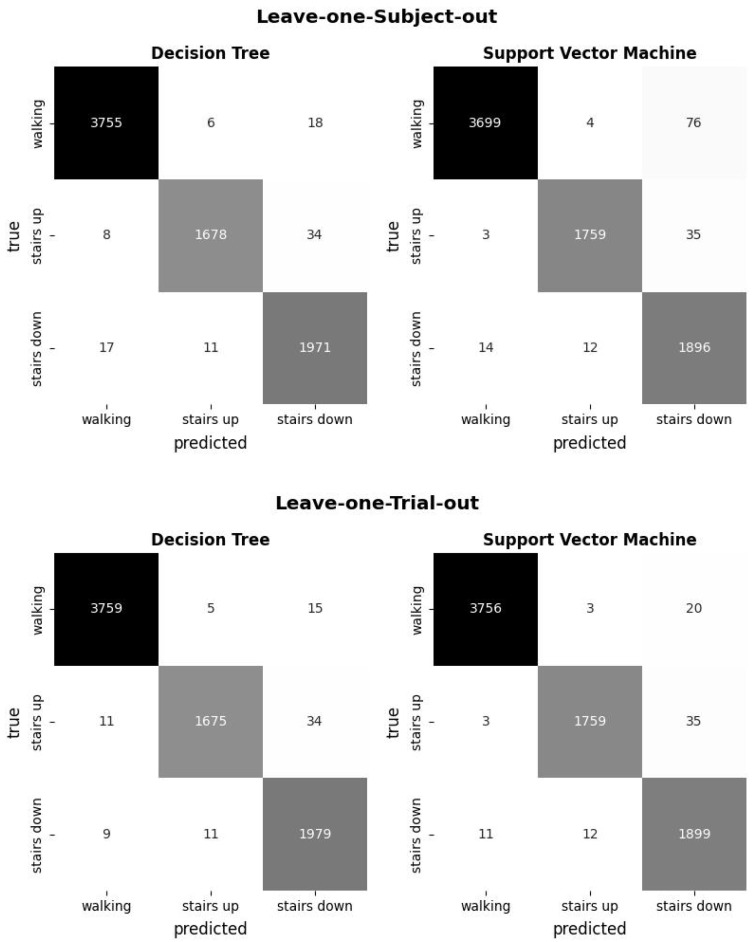
Confusion matrices of the leave-one-subject-out and leave-one-trial-out verification of the Decision Tree model and the Support Vector Machine model.

When evaluating classification models, the confusions, i.e., which class is mistaken for another by the classifier, can indicate which classes are difficult to distinguish for the model. The confusion matrix in Figure 5 shows the sum of these confusions in a 3 × 3 matrix. The column of the matrix indicates the predicted class, while the row indicates the true class. The confusion matrix for each validation is the sum of the confusion matrices of all runs. For both models, the confusion matrix shows that the stairs-up and stairs-down classes are commonly confused. The SVM model also showed common confusion between ‘walking’ and ‘stairs down’ in the ‘Leave one Subject out’ verification, with 76 false classifications registered. This might be an indication of underfitting for a specific physical type of subject. However, this may also be a result of the unequal distribution of training classes, with the ‘walking’ class represented twice as much as ‘stairs up’ and ‘stairs down’ classes.

### 3.2. Map Construction

To create a map using the classifications, the classification results must be geographically referenced. GPS is used to obtain the global location. This implies that the data acquisition must be performed by a subject in an environment with good quality GPS reception. To acquire the data, the SensAA mobile biomechanical data acquisition system is used [15]. The GPS location was captured by the smartphone with the SensAA Android application running on a Samsung Galaxy^®^ S22 smartphone, Samsung Group, Suwon-si, Republic of Korea. The Android operating system limits the frequency at which GPS locations are received. Since there may not be a location for each step the user takes, the GPS locations are interpolated to the timepoints of each step. To assess the quality of data, a dataset was recorded with SensAA. Classification and geographic referencing were performed, and the results are plotted on a map. The results were stored on a Postgres Database with the PostGIS extension installed, which provides geographic functionality. The QGIS geographic information system version 3.22 was used to render and inspect the results.

**Figure 6:**
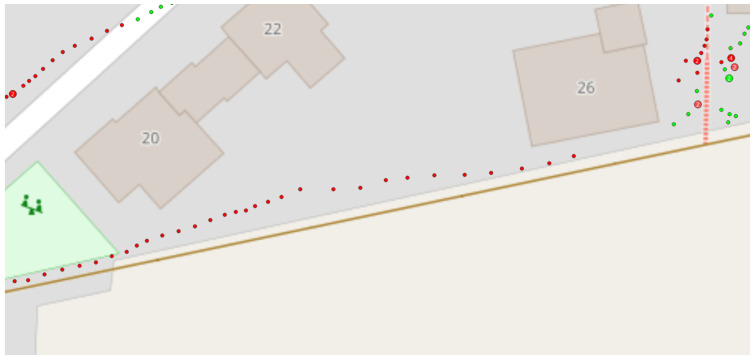
Classified steps at their locations, rendered as a point cluster. The red points denote normal walking steps. The green dots denote stair-climbing steps.

Figure 6 shows the classification results as a point cluster. Multiple points for the same classification are summarized as a larger point. The color of the points indicates the classification result. Steps classified as normal walking are colored red, while stairs-ascending and stairs-descending steps are colored green.

**Figure 7:**
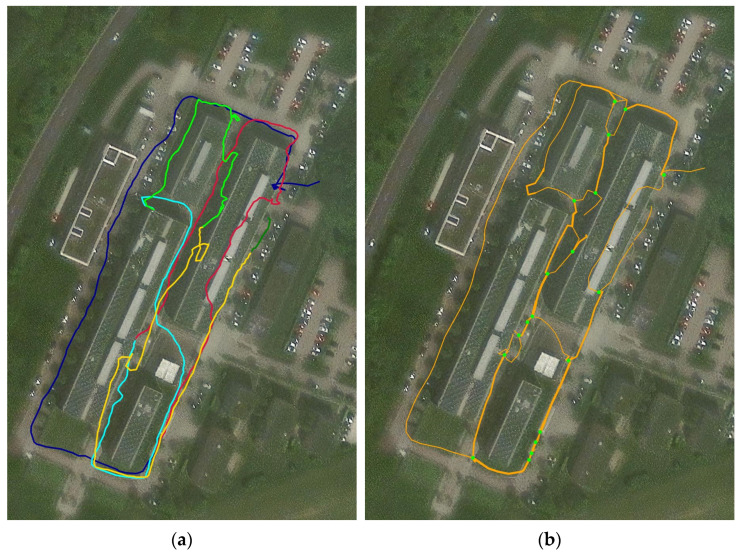
(**a**) Input paths of the map generation test. Each path is denoted in a distinct color. (**b**) The network created by the map construction algorithm. The green dots denote crossings between three or more paths. The thickness of the path illustrates the number of merges from which the path results. Satellite Image source: Esri, Maxar, Earthstar Geographics, and the GIS User Community.

The map construction algorithm proposed in Chapter 2.3 was tested with a simplified model that does not include path types. Instead, each path was considered a simple “trail”. The dataset for testing was recorded on a walk through the Aalen University Campus Burren. The traces were created using a GPS-Tracker app, producing GPS trace files in the gpx format. The GPX files contain multiple “tracks”. From each “track”, a path for insertion into the map was generated. Figure 7a shows the paths to be inserted into the map. Figure 7b shows the resulting map.

In the dataset, we can see some pedestrian walkways and roadways around the campus that were walked one or more times. When the same path was walked more than one time, the traces did not perfectly cover each other, for multiple reasons:

- The user does not walk the same way at the exact same location every time;
- Like all measurements, the recorded GPS location is subject to noise;
- Signal reflections from high buildings can lead to significant deviations.

The red path shows a good example of GPS reflection. While most of the path seems accurate within a few meters, one section of the path completely drifts into the building. The other paths seem to have a high accuracy, for the most part of less than two meters. The results in Figure 7b indicate that the algorithm is working as expected and produces results, even with imperfect input information. In the figure, the number of merges a path is resulting from is illustrated by its thickness. This can be observed as well for the cyan and the yellow path, which resulted in an average path leading between two buildings. The yellow path, which was previously intersecting the building, was pulled back into the pathway. The red path, which was result of GPS reflection, did make it into the map, but was only matched once. With this information, the path can be removed from the map, if it was determined that it was a false measurement based on the lack of merges. The connectors between paths are shown as green dots. Note that only connectors of three or more paths are shown in the figure. Most connectors were created at the expected locations, however some of them seem to be missing.

## 4. Discussion and Outlook

In this work, a cloud-based information system was developed for predictive exoskeleton control and navigation. A concept was introduced for remembering paths typically walked by the user and combining them into a map. By learning properties of the paths, the exoskeleton can pre-emptively prepare for changes in the users’ gait behavior. The path properties are derived from human activity classifications. The classifier developed predicts three different classes with an average F1 score of 97%. The performance is comparable to models already proposed in the literature [16,19]. When evaluating the results, it must be considered that men and women aged 30 to 50 were not represented in the training dataset.

The map construction algorithm was only partly implemented and tested. The results indicate that the concept is viable, and allows small, iterative updates of the map. The future development of the algorithm will include a cleanup logic. This will avoid small path fragments and intersections, as observed in Figure 7b. Furthermore, path properties must still be implemented, and a precise strategy shall be developed for updating path properties when paths are being merged. However, before implementing these optimizations, the stability of the algorithm will be improved so that all connectors are created at the expected locations. The usefulness and accuracy of the path properties are dependent on the robustness of the HAR classifier. Further analysis of these implications is yet to be conducted. Another remaining challenge in map construction is the incorporation of open areas, i.e., plazas. For now, a frequented open area would lead to seemingly arbitrary paths and a very high density of connectors, which could make the map very difficult to navigate through. To mitigate this issue, the algorithm could be extended to detect these places and replace them with a shape matching the outline of the place. The shape could then be connected via connectors to incoming and outgoing paths.

## Figures and Tables

**Table 1:** Results of the performance in terms of precision, accuracy, and F1 score of the Decision Tree model and the SVM model.

	Support Vector Machine			Decision Tree		
	Accuracy	Precision	F1-Score	Accuracy	Precision	F1-Score
	Leave-one-subject-out					
mean	0.98	0.97	0.97	0.99	0.96	0.96
std	0.05	0.08	0.09	0.03	0.10	0.11
min	0.83	0.68	0.63	0.85	0.67	0.63
max	1.00	1.00	1.00	1.00	1.00	1.00
	Leave-one-trial-out					
mean	0.99	0.98	0.98	0.99	0.98	0.98
std	0.03	0.05	0.06	0.03	0.07	0.07
min	0.85	0.68	0.63	0.85	0.67	0.63
max	1.00	1.00	1.00	1.00	1.00	1.00

## Data Availability

The original data presented in the study can be made available on request.
